# Paracentral Acute Middle Maculopathy (PAMM) in Ocular Vascular Diseases—What We Know and Future Perspectives

**DOI:** 10.3390/vision9010019

**Published:** 2025-03-03

**Authors:** Daniele Fumi, Francesco Ruggeri, Davide Fasciolo, Elettra Antonello, Giammarco Burtini, Solmaz Abdolrahimzadeh

**Affiliations:** 1 Ophthalmology Unit, Neurosciences, Mental Health, and Sense Organs (NESMOS) Department, Faculty of Medicine and Psychology, University of Rome Sapienza, 00185 Rome, Italy; 2 St. Andrea Hospital, Via di Grottarossa, 1035/1039, 00189 Rome, Italy

**Keywords:** diabetic retinopathy, hypertensive retinopathy, optical coherence tomography, optical coherence tomography angiography, paracentral acute middle maculopathy, PAMM, retinal artery occlusion, retinal vascular occlusion, retinal vein occlusion

## Abstract

Paracentral acute middle maculopathy (PAMM) is a macular condition primarily detected using optical coherence tomography (OCT) imaging. It presents as hyperreflective bands within the inner nuclear layer (INL) of the retina, often leading to localized degenerative phenomena. PAMM is a condition that reveals a dysfunction in the microvascular network of the retina. However, it is not an isolated phenomenon but rather an indicator of deeper and even systemic, prevalently vascular-related issues related to a wide array of conditions that impact circulation, including retinal vein and artery occlusion, diabetic retinopathy, and hypertensive retinal vascular changes. PAMM occurs due to impaired perfusion within the retinal deep capillary plexus, clinically leading to subtle but noticeable blind spots (scotomas) in the central visual field. Recent advances in imaging technology, particularly optical coherence tomography angiography (OCTA), have provided a clearer view of the underlying vascular alterations. Thus, PAMM may currently serve as a biomarker in broader ocular and systemic pathologies before disease progression. This review explores the latest reports in the literature on PAMM, from its characteristic imaging features to the evolving theories behind its development. By bridging the gap between ophthalmology and systemic health, PAMM may facilitate earlier diagnosis and tailored management strategies for conditions that extend far beyond the eye. Understanding this entity could ultimately transform our approach to assessing vascular health toward further research, risk prediction, and patient care.

## 1. Introduction

**Figure 1:**
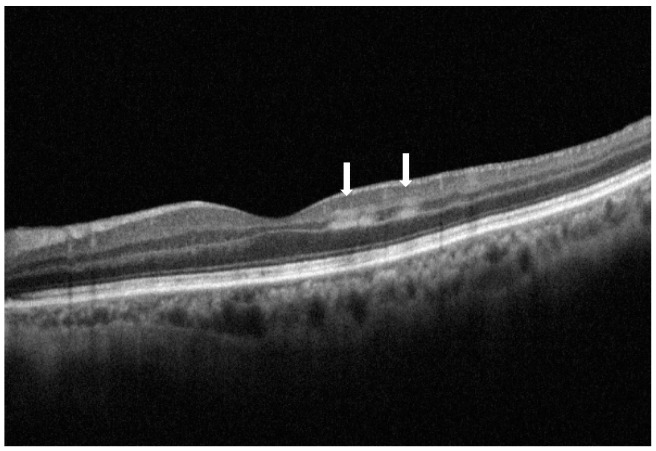
Spectral domain optical coherence tomography cross-sectional image of the left eye. Skip paracentral acute middle maculopathy lesions can be observed in the temporal parafoveal area as two hyperreflective bands prevalently in the inner nuclear layer (white arrows).

Paracentral acute middle maculopathy (PAMM) is a condition that occurs due to macular hypoperfusion [1]. Diagnosis is fundamentally based on optical coherence tomography (OCT), which, in the most common presentation, is recognized by the presence of hyperreflective bands within the inner nuclear layer (INL) without affecting the integrity of the interdigitation zone (IZ) and ellipsoid zone (EZ) [2]. PAMM is caused by impaired retinal capillary system perfusion, leading to infarction of the microvasculature in the acute stage and INL thinning in the chronic phase [3]. Before the advent of OCT, fundoscopic examination in some cases of central retinal vein occlusion (CRVO) showed perivenular whitening [4,5,6] denominated Paques’ plaques [7]. This ophthalmoscopic sign was later identified as PAMM by Sarraf et al. in 2013. differentiating it from acute macular neuroretinopathy (AMN) [8], a rare condition with hyperreflectivity in the outer plexiform layer (OPL) and disruption of the EZ and IZ on OCT. The current use of OCT imaging in routine clinical practice and more refined evaluation of scans has led to enhanced awareness and, consequently, to a prompt diagnosis of PAMM. Figure 1 depicts a case of skip PAMM in its acute phase shown on SD-OCT imaging (Figure 1).

Given the well-established vascular origin of PAMM, a deeper understanding of its pathogenetic mechanisms can provide a more comprehensive framework for a wide range of conditions characterized by vascular involvement, whether primary or secondary. Optical coherence tomography angiography (OCTA) has further enhanced our knowledge, showing that this condition is caused by hypoperfusion prevalently of the retinal deep capillary plexus (DCP) [9]. Early diagnosis of PAMM is critical in detecting early microvascular dysfunction within the retina and in managing ocular vascular diseases, thus reducing the risk of progression to more severe ischemic retinal conditions, such as macular ischemia and retinal vein occlusion. For example, in diabetic retinopathy, the detection of PAMM could indicate the need for further metabolic assessment and glycemic control, measures that could slow down the progression of disease toward advanced forms such as proliferative diabetic retinopathy where visual function would be severely compromised. Early intervention would substantially aim at optimizing systemic vascular risk factors and programming regular retinal examinations in order to mitigate the long-term impact of PAMM on visual function and overall vascular health [2]. The present narrative review will define the distinctive features of this clinical entity within the broader framework of ocular vascular pathologies, investigating its potential contributions to their early detection, clinical intervention, and prognostic assessment.

## 2. PAMM: Multimodal Imaging

**Figure 2:**
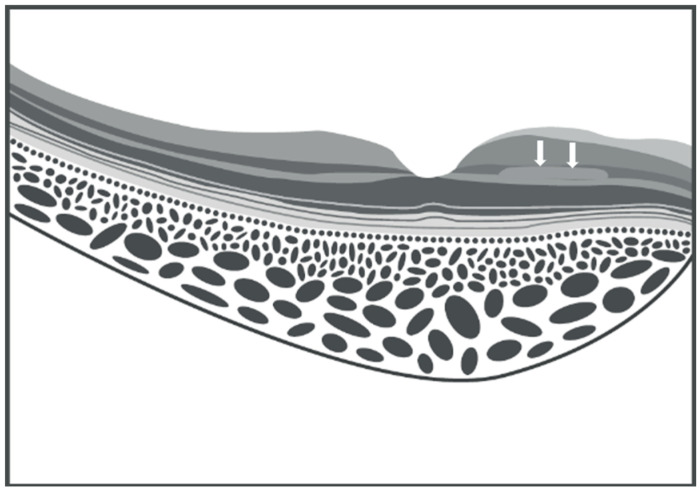
Schematic illustration of paracentral acute middle maculopathy on spectral domain optical coherence tomography. Parafoveal hyperreflective band-like lesion is shown in the inner nuclear layer (white arrows) (graphic illustration courtesy of Dariush Rahimi).

PAMM exhibits a high variety both in its clinical presentation and in the number of pathologies with which it is associated. Therefore, imaging is fundamental for its diagnosis, unequivocally allowing its identification. Spectral-domain optical coherence tomography (SD-OCT) enables rapid, non-invasive imaging, producing high-resolution macular scans with a resolution of about 3–5 μm. The retinal and choroidal structures are visualized in detail; various grades of reflectivity clearly distinguish the individual retinal layers on sagittal and transverse B-scans. The choroid can be assessed using swept-source optical coherence tomography (SS-OCT) [10] and enhanced depth imaging (EDI) technology [11]. This allows for a more precise evaluation of the choroid, which plays a pivotal role in retinal homeostasis, in particular the choriocapillaris—the innermost layer of the choroid—which has been demonstrated to exhibit an increased number of flow voids in patients with PAMM [12]. Figure 2 illustrates PAMM on OCT cross-sectional imaging in its acute phase, with the presence of a hyperreflective band within the INL [13]. This typically progresses towards a thinning, eventually leading to INL atrophy (Figure 2).

**Figure 3:**
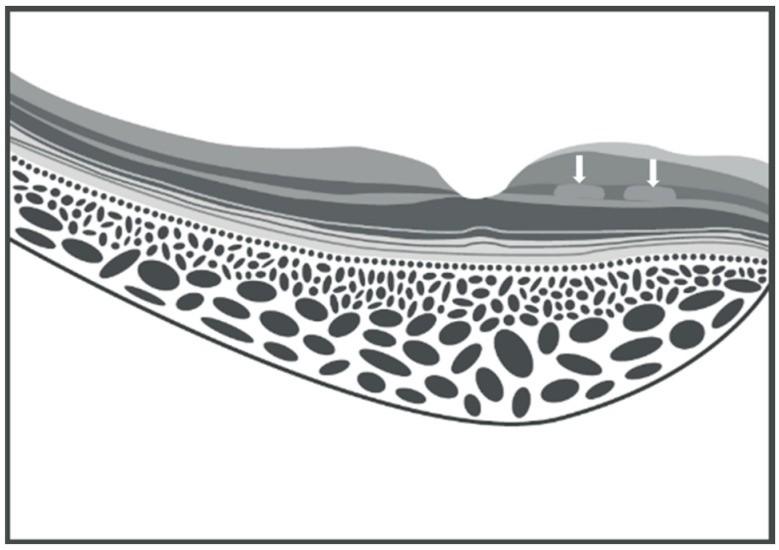
Schematic illustration of skip paracentral acute middle maculopathy on spectral domain optical coherence tomography. Parafoveal hyperreflective band-like lesions with a skip pattern are shown in the inner nuclear layer, as indicated by the white arrows (graphic illustration courtesy of Dariush Rahimi).

**Figure 4:**
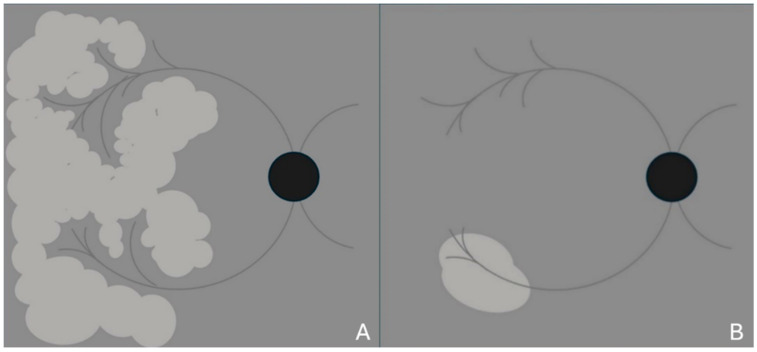
Schematic en-face view illustration of fern-like (**A**) and globular (**B**) paracentral acute middle maculopathy on spectral domain optical coherence tomography.

SD-OCT technology also enables the acquisition of en-face images of the retina and choroid, which are obtained through multiple A-scans. In this manner, it is possible to assess morphological changes in a coronal view, which provides a representation similar to fundus photography [14]. PAMM displays three distribution patterns based on the nature and extent of the ischemic stimulus [15]. The first pattern, termed arteriolar, is discernible through en-face OCT imaging and corresponds to damage characterized by transient occlusion of a retinal arteriole, swiftly followed by circulation restoration. Consequently, the damage is observed at the perivenular areas and appears as lesions defined as skip PAMM in cross-sectional OCT scans (Figure 3), with a fern-like aspect in the en-face view (Figure 4A).

A diffuse and globular form may also be observed if the ischemic stimulus extends more horizontally (Figure 4B). Lesions caused by PAMM typically resolve, resulting in what is known as resolved PAMM [16] or retinal ischemic perivascular lesion (RIPL) [17]. This condition is attributed to the thinning of the INL associated with OPL elevation. It is characterized by a wavy-like profile in the middle retinal layers on cross-sectional OCT scans as a key indicator of resolved PAMM [18]. These post-infarction lesions often manifest as black holes of varying sizes [18] on en-face images. A novel method of evaluating the retinal microvasculature and choriocapillaris is through OCTA technology, where three-dimensional images are constructed by using the intrinsic motion of red blood cells to visualize vascular density and flow area. This method allows evaluation of the retinal superficial capillary plexus (SCP), DCP, and choriocapillaris [19]. Black holes are detected as attenuation of the DCP on en-face OCTA images following ischemic events; the affected vascular area tends to restore itself, leaving few traces of prior damage, except for distinct black holes of memory [20].

Fundus fluorescein angiography (FFA) is an imaging technique performed with the administration of intravenous sodium fluorescein to visualize the retinal vasculature [21]. It enables the evaluation of a plethora of ocular vascular conditions, but it does not allow for the accurate distinction of the deep vascular layer of the retina [22]. Findings in PAMM are not consistent, primarily because there can be many underlying causes but also because FFA cannot discriminate between the DCP and the intermediate capillary plexus (ICP) [23]. Although FFA tends to be normal [24,25,26], it is not uncommon to observe hypofluorescence corresponding to the location of the hyperreflective lesions seen on OCT [27]. It has been hypothesized that the retinal thickening associated with PAMM lesions could be responsible for this FFA presentation, given that once the acute phase has resolved, the hypofluorescence tends to disappear. In cases associated with branch retinal artery occlusion (BRAO), FFA shows a delay in arterial filling in the areas corresponding to PAMM lesions on OCT [13,15,28,29]. Although FFA is not the gold standard for diagnosing PAMM, it remains a valuable tool for identifying underlying conditions that contribute to its development, such as diabetic retinopathy (DR) [30], or for predicting the onset of severe retinal vascular diseases such as retinal vascular occlusion (RVO) or BRAO [31,32,33].

Similarly, near-infrared reflectance images are not always straightforward to interpret. In most cases, parafoveal light-gray areas corresponding to the hyperreflective lesions reported on OCT can be identified [34]. However, there have been reports of cases without any evidence found on near-infrared reflectance imaging [35].

Fundus autofluorescence is another non-invasive imaging method based on the detection of the density of lipofuscin, which is located at the retinal pigment epithelium level. Pathological alterations are distinguished as hyper–hypo and iso-autofluorescent [36]. Although not considered the optimal imaging method to detect PAMM [2], fundus autofluorescence can show fern-like hypoautofluorescence corresponding to the areas of PAMM lesions seen on OCT [37]. Hypoautofluorescence could possibly be due to reduced lipofuscin or ischemia of layers above the RPE.

## 3. PAMM: Clinical Findings

**Figure 5:**
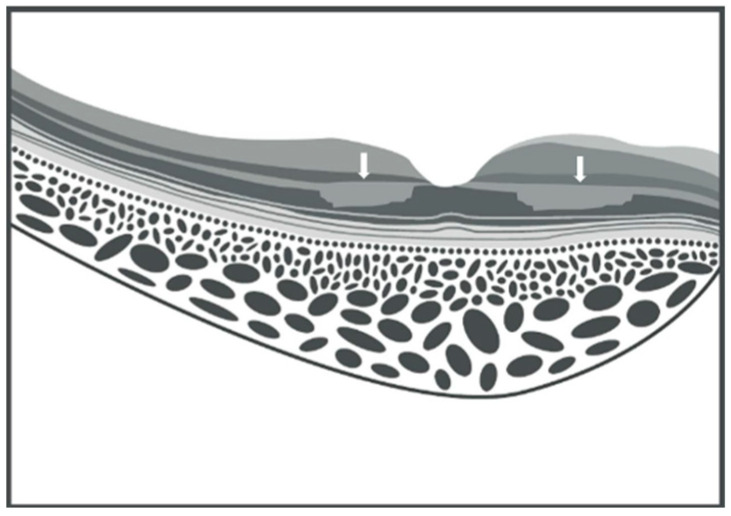
Schematic illustration of acute macular neuroretinopathy on a spectral domain optical coherence tomography. The parafoveal hyperreflective lesions highlighted with the white arrows show the involvement of the outer nuclear layer and the outer plexiform layer (graphic illustration courtesy of Dariush Rahimi).

Single or multiple paracentral scotomas are reported as the typical presentation of PAMM [15]. Clinical symptoms include non-specific central vision blurring and difficulty in focusing on images. The complaint of a fixed gray spot owing to paracentral scotomas has a shape that is strictly dependent on the type and extent of the ischemic area, whereas the scotoma in AMN has been described as having a petaloid, oval, or teardrop shape [38]. Visual acuity in the affected eye may be mildly reduced, with some patients maintaining 20/20 vision [2]. Amsler grid testing is an effective tool for rapidly assessing the scotoma [39]. Microperimetry is another method of identifying the scotoma area as PAMM-mediated damage involving the central retina [40]. This examination can identify regions of decreased differential light sensitivity corresponding to hyperreflective areas observed on en-face OCT images [41]. Fundus examination may appear normal; however, early detection of white-grayish lesions is sometimes reported [5]. These lesions could appear similar to cotton wool spots, but their localization in deeper retinal layers, less distinct association with the distribution of retinal nerve fibers, and whiter coloration can help in differential diagnosis [20]. Before the advent of OCT technology, reported fundus alterations of PAMM were described as ischemic patchy and perivenular whitening of the macular area observed in acute CRVO [4,5,6]. These ophthalmoscopic signs, denominated Paques’ plaques, were subsequently correlated to INL hyperreflective areas on OCT cross-sectional scans a decade later [7]. This OCT correlation was termed PAMM following observation on a larger series of patients [13,35,42]. OCT findings were crucial in defining PAMM as a distinct condition from AMN, a much rarer retinopathy characterized by lesions in the outer plexiform layer (OPL) and in the outer nuclear layer (ONL), accompanied by concomitant disruption of the EZ and IZ [38] (Figure 5).

## 4. PAMM: Pathophysiology and Related Etiologies

Although the pathophysiology of PAMM is not yet fully understood, hypoxia of the intermediate retinal tissue, situated within the watershed zone, an area perfused by the terminal segments of both the choroidal and retinal vasculature, is a key factor [1,43]. Macleod et al. proposed an interesting model to explain the pathophysiological process of PAMM with the theory of the Krogh cylinder model of tissue oxygenation applied to retinal vasculature [1]. In this model, the tissues within the watershed zones are the most vulnerable areas for hypoperfusion damage. The superficial capillary plexus (SCP) does not appear to be directly affected in PAMM, but only secondarily and concurrently with changes in the ICP and DCP. The SCP layer’s dual serial and parallel perfusion might be the reason for the late, if any, involvement in maintaining optimal perfusion despite blood flow supply changes [44]. The DCP, instead, lacks a direct blood supply from the retinal arteries and cannot benefit from descending arterioles from the SCP [45,46]. The only anastomoses that supply the DCP originate from the ICP. Greater clarity regarding the asymmetry of perfusion at this micro-level was achieved by An et al. [47]. Through structural criteria and high-resolution confocal microscopy, these authors reported that the DCP is the only plexus supplied solely by small arterioles, whereas the SCP and ICP are supplied by arterioles with a larger diameter. Furthermore, the small arterioles supplying the DCP are significantly smaller in size compared to the drainage venules. While it is now almost certain that some form of vascular impairment underlies the appearance of PAMM lesions, the specific structures directly affected remain to be elucidated; it is yet to be determined whether the damage occurs at the level of Müller glia, photoreceptors within Henle’s fiber layer, or another structural level [48].

The intraretinal oxygen distribution, in addition to being influenced by local and systemic regulatory factors, exhibits pronounced heterogeneity across individual retinal layers, due to the differing nutritional demands of each individual retinal layer [49]. Another factor in retinal vascular physiology is the presence of distinct oxygen gradients across the retinal layers; the oxygen consumption of each layer is closely linked to the differential oxygen flow. This was demonstrated in studies on animal models by Yu et al., which revealed significantly reduced oxygen tension in the middle retinal layers, approaching hypoxic levels between the SCP and the DCP in physiological conditions [49]. Moreover, the ability to regulate oxygen supply under pathological conditions varies among different vascular structures, with the DCP being the most susceptible to changes in arterial pressure [49], further supporting the role of the DCP in PAMM manifestation. The middle retinal layers may have a higher oxygen demand compared to the inner retina, likely due to the high metabolic activity of horizontal cells [50,51]. This specific vascular configuration makes the area more vulnerable not only to ischemic damage from reduced arteriolar perfusion but also to impaired venous drainage. In a study by Iovino et al. [48], on 17 eyes of patients with concurrent PAMM and AMN, the authors suggested that the pathophysiology behind the two conditions may be similar [35,52]. This finding confirms the close relationship between these clinical entities and supports the hypothesis of a possible pathogenetic continuum. Damage at the intermediate retinal layers is often the first to be identified, sometimes even preceding clinical symptoms. PAMM may be the first sign of a series of vascular events, starting with mild hypoxia that can worsen and affect multiple areas. Over time, the manifestation of PAMM may progress, potentially evolving into more severe conditions like BRVO or CRVO, depending on the extent of retinal damage, known as the retinal ischemic cascade [20]. The aspects of the complex retinal vascular structure underlying the retinal ischemic cascade are illustrated in Table 1 (Table 1).

Ocular vascular factors contributing to PAMM can be grouped into arterial insufficiency, venous impairment, or both. The most common causes are RVO; venous blockage, high outflow pressure, and reduced arterial inflow lead to poor blood flow in the retinal capillaries, particularly at the DCP level [9,51,53]. PAMM occurs in the context of complete or incomplete RVO, more frequently in CRVO but also in BRVO and retinal artery occlusion (RAO), including cilioretinal artery obstruction (CILRAO) or insufficiency [13,54]. Numerous systemic diseases with vascular involvement are also associated with PAMM such as diabetes, hypertension, coagulation disorders, hypercoagulable states, and sickle cell anemia [9]. Other etiologies widely range from medication with vasoactive properties, ocular trauma or surgery, to cardiovascular and vascular surgery [55]. The detailed classification of conditions in which PAMM has been described are shown in Table 2 (Table 2).

**Figure 6:**
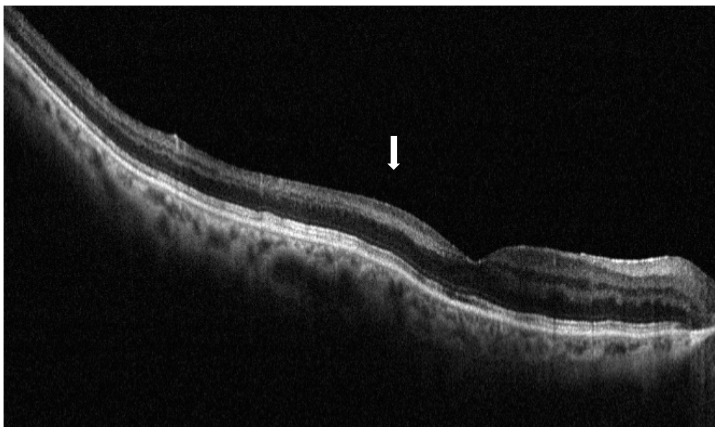
Spectral domain optical coherence tomography cross-sectional scan of the right eye in a patient with a branch retinal vein occlusion history. A longitudinal paracentral acute middle maculopathy lesion can be observed in the temporal parafoveal area as a hyperreflective band in the inner nuclear layer (white arrow).

Since OCT evidence of PAMM can signal a serious ischemic condition as per the ischemic cascade [20], it is important to conduct a thorough medical history evaluation and focused investigations to identify potential systemic risk factors. Indeed, PAMM can be the first sign that precedes clinical symptoms in more extensive lesions such as RVO, RAO, and CILRAO. Figure 6 shows PAMM in an OCT scan of a patient with a history of BRVO [3,20] (Figure 6).

The correlation between minimally ischemic lesions, specifically known as RIPLs (or resolved-PAMM), and the presence of cardiovascular disease was investigated in a retrospective study on 84 patients with cardiovascular conditions and 76 healthy controls [17]. Excluding individuals with RAO, RVO, and DR, the analysis revealed a statistically significant higher mean number of RIPLs in patients versus controls, 2.8 and 0.8, respectively. Additionally, the quantity of RIPLs was positively correlated with higher risk stratifications, as subjects with intermediate and high 10-year atherosclerotic cardiovascular disease risk scores demonstrated a greater number of RIPLs [17].

Rapid diagnostic workup on patients presenting PAMM could potentially aid in making early diagnosis of serious systemic vascular diseases such as cardio and cerebrovascular events [131].

## 5. PAMM in Arterial Insufficiency-Based Retinal Vascular Diseases

Retinal arterial obstruction is an interruption of blood supply to the retinal tissue, not unlike a cerebral stroke, is most commonly embolic, and caused by carotid artery stenosis [132,133,134]. Thrombophilia, vasculitis, vasospasm, giant cell arteritis, and other inflammatory disorders are reported as less common causes [132,133,134,135,136]. The consequent reduction in blood flow results in retinal ischemia or retinal infarction, leading to temporary or permanent visual loss depending on the occlusion site and the duration of blood deprivation [135,136]. RAO is subclassified into three different clinical forms: CRAO, BRAO, or CILRAO, based on the site of occlusion. These forms differ greatly regarding clinical manifestations and overall prognosis [133,134,135]. CRAO is the most clinically significant of the three having the worst overall visual prognosis (5). The visual outcome is generally poor, with visual acuity of 20/400 or worse in 80% of patients with CRAO, whereas in BRAO, visual acuity is 20/40 or worse in 90% of cases [133,134].

Much of what is acknowledged on how PAMM and RAO influence each other is based on the study by Zhang et al. [137] in which 807 eyes of 807 patients were retrospectively analyzed. In total, 252 eyes were positive for RAO (143 CRAO, 105 BRAO, 4 CILRAO), and 49 were positive for both RAO and PAMM. This study assessed the incidence of PAMM in eyes following RAO at a significant rate of 19.44% (49/252). PAMM lesions were found most commonly (32/143 or 22.38%) in the CRAO group [137]. These values were subsequently confirmed in a retrospective investigation by Yu et al., who reported a 22.5% incidence of PAMM in eyes with RAO, although a much smaller sample of 40 patients was considered [137]. Nevertheless, the incidence of PAMM may be underestimated, given the transient nature of lesions and concomitant conditions such as hemorrhages or hyperreflectivity that could make the detection of PAMM lesions more challenging [137]. Interestingly, PAMM was reported in 100% of eyes with isolated CILRAO with or without CRVO, creating the term cilioretinal artery insufficiency. Rather than depending on complete occlusion, this phenomenon was hypothesized to be caused by hypoperfusion of the cilioretinal artery [61].

A retrospective study on a total of 88 patients, 58 with CRAO and 30 with BRAO, of which 52 were also PAMM positive, showed that patients with concomitant PAMM and RAO had better BCVA over time [136]. These findings could justify more proactive management in these circumstances in order to optimize the final outcome [136].

In accordance with the aforementioned report, Gong et al. found that patients with CRAO and concomitant PAMM had better BCVA than those without PAMM [138]. This may suggest that, in the context of CRAO, the presence of simultaneous PAMM, as a self-limiting mechanism, leads to less devastating damage to BCVA compared to conventional CRAO. The authors based this conclusion on OCTA findings, which showed capillaries and small terminal arteries without blood flow signals in patients with PAMM. They suggested that this could be an indicator of the terminal arteries and connected capillaries regulating vascular resistance through the mechanism of constriction and occlusion, thus sustaining a constant blood flow albeit changes in perfusion pressure [138]. As an additional finding, they reported that the optic disc boundary in PAMM-positive patients was more blurred than in PAMM-negative patients. Although observed in both groups, bleeding and cotton wool spots were much more prominent in the PAMM-negative group [138]. Interestingly, macular edema and higher central retinal thickness were more consistently found in the PAMM-negative group, eventually leading to a worse visual prognosis. At OCTA, vessel density (VD) for both the DCP and SCP was lower in the PAMM negative group but did not reach statistical significance [138].

The paucity of studies in the current literature is a limitation on presently considering PAMM as a valid biomarker for predicting structural and functional parameters, such as macular edema or increased macular thickness and visual acuity prognosis, in the context of RAO. Therefore, further studies on broader cohorts of patients and targeted methodology are warranted.

## 6. PAMM in Venous Impairment-Based Retinal Vascular Diseases

Retinal vein occlusion (RVO) is a condition where retinal vein circulation is impaired. CRVO occurs when the occlusion is localized within or posterior to the optic nerve head, whereas any obstruction involving a tributary branch is classified as BRVO. RVO constitutes a major cause of blindness, second only to DR [139,140]. Atherosclerosis and related risk factors are strongly correlated to RVO, but other processes, such as vasospasm, compression, and inflammatory factors, also play a role. Arteriosclerosis, systemic hypertension, and diabetes are the most common among the many risk factors reported for BRVO [141,142]. There is a high risk of vision loss in patients with RVO owing to complications arising from disrupted blood flow, ischemia, and macular edema [143]. Indirect ophthalmoscopy, FFA, and OCT are fundamental tools for confirming the diagnosis, assessing neovascular risk, and evaluating macular edema [144]. OCT is capable of detecting signs of ischemia in the INL, including AMN, PAMM, and prominent middle limiting membrane (p-MLM) [144].

A study by Bakhoum et al. on patients with acute RVO highlighted that PAMM with the perivenular fern-like feature is correlated with a better BCVA compared to PAMM with a globular pattern. Eyes with more severe vascular damage, exhibiting ischemia in both the central and inner retina, demonstrated the lowest BCVA. The final visual acuity was lower in patients with globular PAMM or ischemia of the inner retina [53]. We could suggest that these results strengthen the hypothesis of the increasing damage induced by more severe forms of PAMM in the context of the already mentioned retinal ischemic cascade.

Maltsev et al. analyzed the prevalence of resolved PAMM in the fellow eyes of patients with unilateral RVO and compared these with the eyes of healthy subjects in order to assess whether there was an association between resolved PAMM lesions and RVO [18]. OCTA can detect acute PAMM through the presence of blood flow signal voids in the DCP [145]. Indeed, in this study, OCTA images of the DCP in resolved PAMM lesions of large dimensions showed flow signal voids/attenuations, whereas smaller lesions primarily exhibited normal or slightly attenuated flow signals [18]. OCTA alone, in asymptomatic patients, might not be sufficient to detect all resolved PAMM lesions, since potential reperfusion of the DCP has been described [145,146]. It was reported that in the acute phase of focal forms, blood flow at the DCP level remained intact, though a progressive attenuation was observed in the chronic phase. In contrast, more diffuse forms exhibited a more pronounced reduction in flow signal during the chronic phase, potentially leading to the permanent loss of the DCP [146]. As previously mentioned, PAMM is not an independent clinical entity but rather a manifestation of an underlying pathology. Depending on the extent of the ischemic insult, the morphological presentation follows a progressive pattern, initially affecting only the perivenular region in mild forms. In severe forms the ischemia has a diffuse horizontal progression through the INL, and can extend vertically involving the inner retina in the most severe occlusive events [9].

In a recent article, Cabral et al. analyzed the retinal capillary plexuses using High-Resolution OCT-A in order to compare cross-sectional OCT characteristics with volume rendering of vascular network [147]. This provided additional insight into the DCP drainage system and its interplexus connections, which is often implicated in various macular vascular pathologies, including RVO [147]. Interestingly collateral vessels following RVO develop preferentially in the DCP [148]. This is primarily driven by the low perfusion pressure and inflow velocity [149], as well as distinct histological properties of the DCP vessel walls, which exhibit increased compliance and distensibility [47]; this could possibly be implicated in the transient or permanent nature of PAMM.

Maltsev et al. evaluated OCT structural features, such as thinning of the INL associated with elevation of the OPL, as key indicators of resolved PAMM. These authors reported resolved PAMM lesions in 71.1% of patients (32/45) with BRVO and in 71.4% of patients (15/21) with CRVO [18]. In healthy patients, these lesions were found in 19.3% of individuals (11/57), especially in the elderly population, patients with hypertensive disease, and smokers [18]. However, in the fellow eyes of patients with RVO, the prevalence of such lesions was significantly higher than that found in age-matched healthy controls [18]. These data suggest that resolved PAMM lesions have an association with RVO and could represent a risk indicator for the severe form of retinal vascular events.

In a recent investigation, Cabral et al. reported the presence of a novel biomarker in retinal vascular occlusive diseases described as hypointense SS-OCTA bands on en-face images of the superficial and deep vascular plexus [150]. The authors studied nine patients with partial RVO or nonischemic RVO and found a correlation between the extent of these hypointense bands and delayed artero-venous filling shown on FFA. Although they observed PAMM in 78% of patients, these lesions did not persist in the eyes continuing to show hypointense bands at long-term follow ups. The authors suggested that the slow flow, beneath the limit of detection of SS-OCTA devices, may not always be enough to cause PAMM on structural OCT, allowing for the potential distinction between ischemic and non-ischemic forms [150].

Zhang et al. [151] analyzed the clinical features of PAMM and its correlation with p-MLM in patients presenting RAO and RVO. This sign is identified as a hyper-reflective line in the inner synaptic portion of the OPL in cross-sectional OCT images. p-MLM appears to develop due to swelling of the cytoplasm localized in the synaptic areas of the OPL and is observed in both RAO and RVO [152,153,154]. PAMM and p-MLM generally manifest in the paracentral area, often extending further outward [151]. The p-MLM sign can aid in differentiating the ischemic and non-ischemic forms of RVO but is also found in RAO [151]. p-MLM and PAMM share a similar pathogenesis; in the presence of mild retinal hypoperfusion, the entire inner synaptic OPL and adjacent INL tissue develop ischemic phenomenon leading to edema, shown on OCT as the p-MLM sign (continuous hyperreflective line) and skip PAMM, respectively [151]. Recently, it was reported that OCT can detect p-MLM in 94% of ischemic versus 66% of non-ischemic RVO [154]. However, according to Zhang et al., these percentages were significantly lower: in 78 eyes presenting PAMM, only 30.77% (24 eyes, of which 7 RVO and 17 RAO) exhibited p-MLM. In seven eyes with RVO and the p-MLM sign, skip PAMM lesions were present, whereas, in RAO, p-MLM was associated with all types of PAMM lesions. Additionally, patients with p-MLM tended to be significantly older [151]. The incidence of PAMM was reported to be higher in eyes with RAO and less frequent in eyes with RVO occurring at a rate of 19.44% and 5.23%, respectively [151]. Furthermore, BCVA was better in patients with RVO rather than RAO [151]. In alignment with these findings, Limoli et al., in a retrospective study on 78 patients presenting PAMM (16 RAO, 20 RVO, and 40 PAMM not associated with retinal vascular diseases), found that baseline BCVA in PAMM patients with RAO was significantly worse than those with RVO or those with no retinal vascular disease [155]. While the studies by Zhang et al. [137] and Limoli et al. [155] provide valuable insights into different aspects of PAMM, it should be noted that these studies differ significantly in terms of sample size, patient cohorts, and experimental design. As such, direct comparisons between their findings may not be entirely accurate. A formal meta-analysis would be necessary to perform a rigorous comparison. In the present discussion, these comparisons are presented primarily to highlight trends and findings rather than to draw definitive conclusions.

In line with previous observations regarding the retinal vascular microstructure, the higher presence of PAMM in RAO compared to RVO can be analyzed using the concept of arteriolar–venular asymmetry documented at the DCP level. The difference in the incidence of PAMM and its variable structural morphology in RVO and RAO is a challenging area of research worthy of further investigation. Future studies may elucidate the clinical implications of PAMM features and their significance in ocular vascular disease and regarding the use of these lesions as a potential biomarker of risk.

## 7. PAMM in Combined Vascular Deficiency-Based Retinal Vascular Diseases

### 7.1. Diabetic Retinopathy

Diabetic retinopathy represents the most significant complication associated with diabetes mellitus and is a main cause of vision impairment in adults [156]. This condition can be classified into two distinct forms: non-proliferative diabetic retinopathy (NPDR), which constitutes the early stage of the disease marked by retinal alterations such as microhemorrhages, exudates, and microaneurysms, and proliferative diabetic retinopathy (PDR), where ischemia-induced stimuli result in the formation of neovascularization [157]. Diagnosis is predominantly clinical, relying on the identification of these retinal findings through fundoscopy, FFA, and OCT. The primary contributor to vision loss in DR is the onset of diabetic macular edema, which can develop at any stage of disease [158].

The assessment of capillary plexuses in diabetic patients has been the focus of numerous studies [22,159,160]. In a study by Hwang et al. [161], utilizing projection-resolved OCTA (PR-OCTA), the capillary network was segmented into three distinct plexuses. The study revealed significant vessel dilation characterized by hairpin loops in the deep plexuses, particularly within regions of hypoperfusion. These vascular changes demonstrated a strong correlation with advanced stages of DR [161]. Ashraf et al. conducted a study utilizing PR-OCTA to assess the differences in the three capillary plexuses on 396 eyes with varying stages of DR and found that the VD within the capillary plexuses showed distinct correlations with DR severity. Specifically, the DCP demonstrated a strong association with the progression from no DR to mild DR, whereas the SCP exhibited the most significant correlation with increasing severity within NPDR. Both the SCP and DCP were found to have significantly reduced VD in cases of PDR [162].

The correlation between PAMM and DR was investigated by Maltsev et al. in a study analyzing macular OCT scans from 51 patients with DR, categorized into three progressive severity levels: mild NPDR, moderate-to-severe NPDR, and PDR. The study identified a statistically significant trend of an increasing prevalence of resolved PAMM lesions corresponding to the increasing severity of DR. Moreover, linear regression analysis revealed, after adjusting for the confounding factors of hypertension and age, that DR severity remained independently correlated with the presence of resolved PAMM lesions [67]. Additionally, OCTA analysis of the three capillary plexuses showed that eyes with resolved PAMM lesions had a reduced VD in the DCP compared to those without such lesions [67].

These findings underscore the necessity of evaluating the SCP and DCP independently, as each plexus is differentially implicated in the progression of DR severity. PAMM in DR is worthy of further investigation, especially in view of the higher presence of RIPLs in different DR stages, potentially considering the use of resolved PAMM as an indicator of risk for major ocular vascular events in diabetic patients.

### 7.2. Hypertensive Retinopathy

Hypertensive retinopathy is one of the potential complications of arterial hypertension [163]. The persistent elevation in blood pressure can damage multiple organs, a phenomenon known as hypertension-mediated organ damage, primarily attributed to underlying vascular alterations [164]. In hypertensive retinopathy, retinal microvascular damage [165] increases the risk of retinal occlusive events [166].

Several studies correlated PAMM lesions with both retinal and systemic vascular morbidity. A case–control study specifically focusing on hypertensive pathology was conducted by Burnasheva et al. This study included 27 patients with mild hypertension and 24 healthy controls [16]. Although there were no significant differences in the VD of the SCP and DCP, the authors reported that chronic PAMM lesions were present in 88.9% of patients with arterial hypertension, compared to only 16.7% in healthy subjects [16]. The authors demonstrated with high statistical confidence that the presence of PAMM was almost exclusive to patients with mild hypertension. In line with the retinal ischemic cascade theory, they suggested that PAMM may represent the initial damage to the retinal microvasculature, even in chronic conditions such as hypertension [16].

Both in DR and hypertensive retinopathy, resolved PAMM has thus shown to play a role in prognostic risk stratification better than the evaluation of the DCP alone. This, on one hand, confirms the microvascular recovery that occurs during hypoperfusion events that lead to PAMM, and on the other hand, it might highlight the better sensitivity of RIPL in documenting such events.

## 8. Conclusions and Future Perspectives

The continuous evolution of imaging techniques, particularly OCT and OCTA, has enabled the refined characterization of even minimal macular alterations, leading to the current definition of PAMM as an autonomous entity. Thus, this distinct clinical sign has been analyzed in the wider frame of numerous ocular and systemic diseases, but also in healthy, or apparently healthy, subjects. Inevitably, interest in PAMM has grown, allowing, with progressive ongoing research, a better definition at the pathophysiological level in the context of the hypothesized retinal ischemic cascade. Germane to this novel evidence, it is becoming increasingly clear that PAMM may represent both an early diagnostic tool in a widespread group of ocular vascular pathologies and a biomarker for better stratifying prognosis and treatment in the future.

The major limitation of the present review is the lack, in the literature, of large population-based studies regarding each specific pathology where PAMM is encountered. Furthermore, at present few studies have analyzed the exact relationship between the presence of PAMM and vascular risk. It is reasonable to hypothesize that PAMM could play a more central role in decision-making processes across various diseases, primarily or secondarily affecting the retinal microvasculature. Recent studies have evaluated the ability of machine learning software to assess changes such as RIPLs from small datasets, showing that in just six hours of training, lesions could be identified with high accuracy (90%), specificity (93%), and negative predictive value (95%) [167]. This demonstrates how modern artificial intelligence-driven software can assist in identifying this clinical entity. The close relationship between PAMM and systemic vascular alterations, the severity of which appears to increase with the number of resolved PAMM lesions, opens the possibility of systemic risk stratification through their quantification. Through longitudinal studies, it may become possible to develop an algorithm that can accurately correlate the number of resolved PAMM lesions with the probability of a major ischemic event, thereby enabling an increasingly tailored therapy, and optimizing management timelines.

## Figures and Tables

**Table 1:** The retinal ischemic cascade–the underlying retinal pathophysiological features.

Pathophysiological Processes in PAMM	Key Points
Hypoxia in the Intermediate Retinal Tissue	The intermediate retinal tissue, within the watershed zone, is hypoxic due to insufficient perfusion by terminal segments of choroidal and retinal vasculature.
Watershed Zone Vulnerability	Based on the Krogh cylinder model, tissues in watershed zones are more susceptible to hypoperfusion damage.
Capillary Plexus Dynamics	- The SCP is not directly affected, likely due to its dual serial and parallel perfusion. - The DCP lacks direct arterial supply and is solely dependent on the ICP for perfusion.
Structural and Microvascular Vulnerability	- The DCP is uniquely supplied by small arterioles, which are smaller than the venules draining them. - This asymmetry increases the DCP vulnerability to ischemia and venous drainage issues.
Intermediate Retinal Layer Susceptibility	The middle retinal layers have higher oxygen demand due to horizontal cells metabolic activity, making them prone to ischemic damage.

Abbreviations: deep capillary plexus (DCP); intermediate capillary plexus (ICP); superficial capillary plexus (SCP).

**Table 2:** Paracentral acute middle maculopathy-related etiologies.

**Ocular Vascular Factors**	
Venous impairment	Retinal vein occlusion [18,35,56,57]
Arterial insufficiency	Incomplete central retinal artery occlusion [58]
	Incomplete cilioretinal artery occlusion [35,59,60,61]
	Branch retinal artery occlusion [62,63,64,65]
	Reperfused central retinal artery occlusion [66]
	Ocular ischemia syndrome [34]
Combined vascular insufficiency	Diabetic retinopathy [13,34,43,67]
	Radiation retinopathy [68]
	Hypertensive retinopathy [16,34]
	Sickle cell retinopathy [34,69]
**Other Ocular Factors**	
Ocular trauma	Orbital compression injury [34]
Ocular surgery complications	Post-cataract [70,71]
	Post-vitrectomy [43,72,73]
	Macular pucker peeling [74]
	Pterygium surgery [75,76]
	Cosmetic iris color change laser [77]
Glaucoma	Primary angle closure glaucoma [78]
Optic nerve disorders	Anterior arteritic ischemic optic neuropathy [79]
	Anti-Mog positive neuritis [80,81]
Congenital diseases	Foveal hypoplasia [82]
	Primary congenital glaucoma [24]
Uveitis	Intraocular tubercolosis [83]
	Idiopathic retinitis, vasculitis, aneurysms, and neuroretinitis syndrome [84]
	Human immunodeficiency virus retinopathy [85]
**Systemic Vascular Factors**	
Venous return impedance	Chronic obstructive pulmonary disease [54]
Arterial flow insufficiency	Carotid stenosis [34,86,87]
	Ischemic cardiomyopathy [88]
	Cardiac arrest [89]
	Sickle cell disease [34,63,90]
	Anemia + Kikuchi-Fujimoto disease [8]
	Polycythemia [91]
	Aorthic aneurism [92]
	Tetralogy of Fallot [91]
**Other Systemic Factors**	
Infections	Coxsackie A4 infection [93]
	COVID-19 [45,81,82,83,84,94,95,96,97]
	Post-viral purtscher-like retinopathy [98,99]
Congenital diseases	Neurofibromatosis Type 2 [100]
Immune-mediated diseases	Giant-cell arteritis [101]
	Eosinophilic granulomatosis with polyangiitis [102]
	Juvenile dermatomyositis [103,104]
	Susac syndrome [105]
	Primary antiphospholipid syndrome [106,107]
	Livedo reticularis [62]
	Ulcerative colitis [108]
	Amyloidosis [37]
	Leukocytoclastic vasculitis [57]
**Neurological Disorders**	Migraines [109,110]
	Leukoencephalopathy [111]
	Idiopathic intracranial hypertension [112]
	Meningitis [113]
**Trauma**	Purtscher retinopathy [34,114,115,116]
	Blunt trauma [117]
**Iatrogenic Factors**	
Surgery	Aortic aneurism repair [118]
	Cardiac catheterization [119]
	Cardiopulmonary bypass [55]
	Cosmetic filler injections [120,121]
	Embolic events after septoplasty [122]
	Endovascular embolization [123]
Medications	Caffeine consumption [8]
	Oral contraceptives [34,124]
	PDE-5 inhibitors [61,125]
	Pembrolizumab [126]
	Synephrine [29]
	Sumatriptan [8,127]
Vaccinations	H1N1 vaccination [34]
	Hepatitis B vaccination [128]
	COVID-19 vaccination [129]
**Idiopathic**	
	In otherwise healthy individuals [42]
	Pregnancy [130]

## Data Availability

No new data were created or analyzed in this study. Data sharing is not applicable to this article.
